# Encapsulation of a TRPM8 Agonist, WS12, in Lipid Nanocapsules Potentiates PC3 Prostate Cancer Cell Migration Inhibition through Channel Activation

**DOI:** 10.1038/s41598-019-44452-4

**Published:** 2019-05-28

**Authors:** G. P. Grolez, M. Hammadi, A. Barras, D. Gordienko, C. Slomianny, P. Völkel, P. O. Angrand, M. Pinault, C. Guimaraes, M. Potier-Cartereau, N. Prevarskaya, R. Boukherroub, D. Gkika

**Affiliations:** 1 Univ. Lille, Inserm, U1003 - PHYCEL - Physiologie Cellulaire, F-59000 Lille, France; 2 Laboratory of Excellence, Ion Channels Science and Therapeutics, Villeneuve d’Ascq, France; 3 Univ. Lille, CNRS, Central Lille, ISEN, Univ. Valenciennes, UMR 8520, IEMN, F-59000 Lille, France; 4 Univ. Lille, U908 - CPAC, Cell Plasticity and Cancer, F-59000, Lille, France; 5 CNRS, CPAC, Cell Plasticity and Cancer, Lille, France; 6 Université de Tours, Nutrition, Croissance et Cancer, Inserm UMR1069, Tours, France; 7 Ion channel Network and Cancer-Canceropole Grand Ouest, (IC-CGO), Nantes, France

**Keywords:** Cell migration, Nanobiotechnology

## Abstract

In prostate carcinogenesis, expression and/or activation of the Transient Receptor Potential Melastatin 8 channel (TRPM8) was shown to block *in vitro* Prostate Cancer (PCa) cell migration. Because of their localization at the plasma membrane, ion channels, such as TRPM8 and other membrane receptors, are promising pharmacological targets. The aim of this study was thus to use nanocarriers encapsulating a TRPM8 agonist to efficiently activate the channel and therefore arrest PCa cell migration. To achieve this goal, the most efficient TRPM8 agonist, WS12, was encapsulated into Lipid NanoCapsules (LNC). The effect of the nanocarriers on channel activity and cellular physiological processes, such as cell viability and migration, were evaluated *in vitro* and *in vivo*. These results provide a proof-of-concept support for using TRPM8 channel-targeting nanotechnologies based on LNC to develop more effective methods inhibiting PCa cell migration in zebrafish xenograft.

## Introduction

In developed countries, prostate cancer (PCa) is the second most frequently diagnosed cancer and the third most common cause of death by cancer in men^1^. More specifically, the development of metastasis during the late stages of PCa is the main cause of cancer-related mortality and is dependent on two key processes: cell migration by cancer cells that invade adjacent tissues and their subsequent intravasation into blood/lymphatic vessels and tumor vascularization. Cell migration is an essential step that leads to the development of metastasis, and several studies have implicated the transient receptor potential melastatin member 8 (TRPM8) channel in PCa migration processes^2–4^. Indeed, these studies suggest that TRPM8 plays a putative protective role in metastatic PCa progression because enhancing the expression and/or activation of this channel blocks PCa cell migration^3–5^. Due to its effects on migration, the activation of TRPM8 seems to exert a protective anti-invasive effect and could therefore be a particularly promising therapeutic target.

Because of their localization in the plasma membrane, ion channels, such as TRPM8 and other membrane receptors, are promising pharmacological targets. Recent advances in pharmacology and biotechnology use nanocarriers to deliver pharmacological compounds by targeting plasma membrane proteins^6^. In this way, nanocarriers targeting membrane receptors, such as HER2 and EGFR^7,8^, or ion channels using curcumin, a TRPA1 activator^9^, have been developed and used in the context of breast or prostate cancer treatment. Nanocarriers have been developed and are used in several forms, including as liposomes, virosomes, solid lipid nanoparticles, polymeric nanoparticles or protein conjugates, depending on the application^10^. In this study, lipid nanocapsules (LNC600) were prepared according to Heurtault *et al*.^11^. The LNC600 were composed of an oily liquid core (Labrafac WL 1349) surrounded by a mixed layer of lecithin (Phospholipon G90) and a hydrophilic surfactant (Solutol HS15, a polyethylene glycol chain with a molecular weight (MW) of 600 Dalton and containing an average of 15 ethylene glycol units). There is strong interest in water-dispersible nanocarriers in drug delivery, and they appear to be useful nanostructures for the solubilization of hydrophobic drugs.

Many chemical compounds are able to activate TRPM8. The most extensively studied of these are menthol, eucalyptol and chemicals that elicit cool sensations^12^, but other substances do exist, such as the supercooling icilin and the selective agonists WS12, CPS-369 (two menthol derivatives) and D-3263^12–18^. Experimental evidence has shown that the activation of TRPM8 by different compounds involves different mechanisms and binding sites in the TRPM8 channel, with these differences depending on the chemical structures of the compounds and TRPM8. This channel, like all other TRP channels, has 6 transmembrane (TM) domains with cytoplasmic N and C-termini, and the functional channel is formed by a homotetrameric assembly comprising four subunits^19–21^. With regard for the distinct mechanisms by which TRPM8 activation is induced by different compounds, it has been demonstrated that icilin-mediated TRPM8 activation depends on intracellular Ca^2+^ levels, whereas it can be activated by menthol, CPS-368 or WS12 in the absence of intracellular Ca^2+^ ^13,15^. Despite their different mechanisms of activating TRPM8, these chemical compounds generally serve as positive allosteric modulators. More specifically, because the activation of TRPM8 is also voltage-dependent, agonists shift the activation threshold towards more negative potentials, whereas antagonists exert their effect by shifting the voltage dependence of TRPM8 activation towards more positive potentials^22–24^.

The aim of this study was to use nanocarriers encapsulating a drug to efficiently activate TRPM8 channels to arrest prostate cancer cell migration. To achieve this goal, the most efficient TRPM8 agonist, WS12, was encapsulated into LNC600. The effect of the nanocarriers on channel activity and cellular physiological processes, such as cell viability and migration, were evaluated *in vitro* and *in vivo*.

## Results

### Encapsulation of WS12 in lipid nanocapsules allows the use of lower agonist concentration for TRPM8 activation

**Figure 1:**
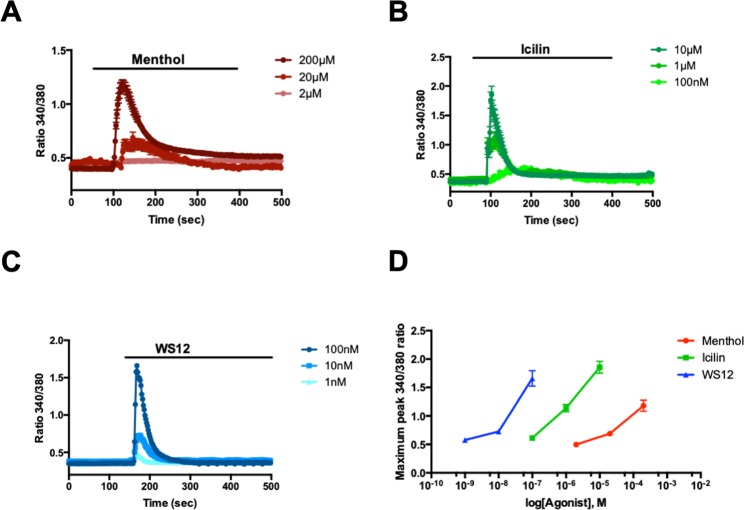
The effects of TRPM8 agonists on channel activity. Averaged time courses of PC3-M8 cell cytosolic Ca^2+^ concentrations (expressed as the f/f0 ratio; mean ± SEM) in response to (**A**) menthol (200 µM, 20 µM and 2 µM), (**B**) icilin (10 µM, 1 µM and 100 nM) and (**C**) WS12 (100 nM, 10 nM and 1 nM). (**D**) Representative dose-dependent curve of TRPM8 activation following treatment with three concentrations of menthol, icilin and WS12. N = 3 independent assays, and data represent 150 cells per condition.

We tested the three best-known TRPM8 agonists, menthol, icilin and WS12, to determine which is the most efficient drug when encapsulated in LNC600. To this end, we used calcium imaging to measure TRPM8 activation in prostate cancer cells (PC3) that overexpressed the channel after the cells were stimulated with the three agonists at concentrations used in previous studies^3,4,25,26^: a high dose corresponding to 200 µM of menthol, 10 µM of icilin and 100 nM of WS12 as well as two lower concentrations, including 1/10 (medium dose) and 1/100 (low dose) of the most effective drug (Fig. 1). First, we show that treatment with 200 µM and 20 µM but not 2 µM menthol induced an increase in TRPM8-mediated intracellular Ca^2+^ (Fig. 1A). With regard for icilin, TRPM8 activation was induced by 10 µM and 1 µM icilin and, to a lesser extent, by a lower dose of 100 nM icilin (Fig. 1B). Finally, TRPM8 was activated by WS12 in a dose-dependent manner with the maximum activation achieved by 100 nM WS12 and a lesser degree of activation attained by 10 nM and 1 nM WS12. When we compared the dose response curves of the three agonists, we found that WS12 appeared to be the most effective because it activated TRPM8 at 100-fold and 2000-fold lower concentrations than icilin and menthol, respectively (Fig. 1D). TRPM8 responded in a dose-dependent manner to menthol, icilin and WS12 at minimal activating concentrations of 2 µM, 100 nM and 1 nM, respectively. Given the comparatively lower concentration of WS12 needed to achieve an equivalent activation of TRPM8 (nanomolar range: 10^4^ less than menthol and 10^2^ less than icilin) (Fig. 1), WS12 appeared to be an interesting candidate for encapsulation in LNC600.

**Figure 2:**
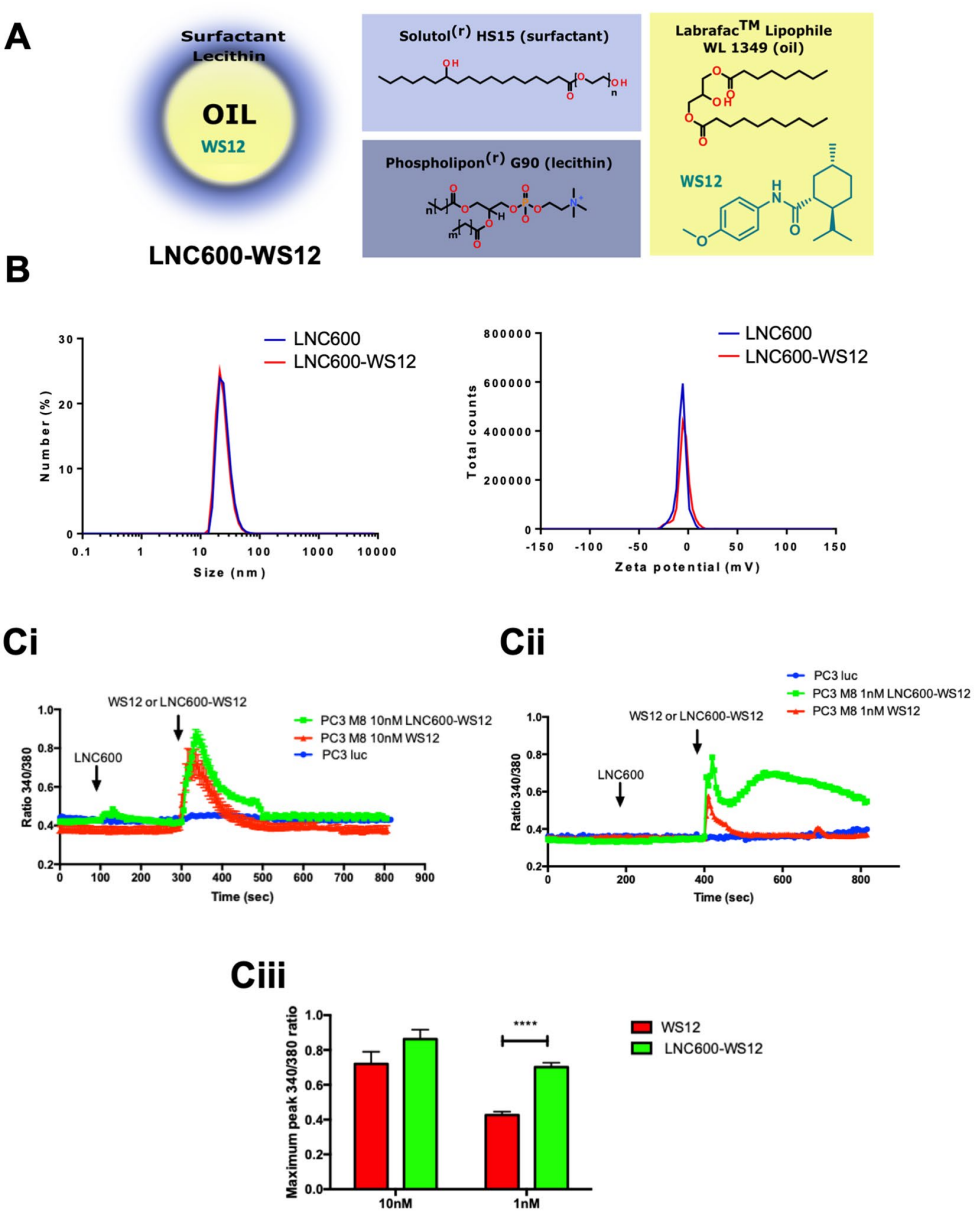
Encapsulation and action of the highly efficient TRPM8 agonist, WS12, following encapsulation into lipid nanocapsules. (**A**) Schematic diagram illustrating the formulation of lipid nanocapsules (LNC) containing WS12 (left panel) as well as the molecular structures of LNC components (right panel). (**B**) Characterization by DLS of LNC600 and LNC600-WS12 (size, left panel; zeta potential, right panel). (**C**) PC3 (wt) and PC3-M8 cells were used for calcium imaging to study the activation of TRPM8 following the application of different concentrations of free WS12, empty nanocapsules (empty LNC600) and the encapsulated form of WS12 (LNC600-WS12). Cells were treated with 10 nM (**Ci**) or 1 nM (**Cii**) of empty LNC at 100 s followed by treatment with free or encapsulated form of WS12 at 300 s. Bar graphs (**Ciii**) represent the maximum values of the 340/380 ratio of Fura-2AM probes induced by TRPM8 activation in cells treated with free or LNC600-WS12 at 10 and 1 nM. N = 3 independent assays, data represent 200 cells per condition. ****P < 0.0001 (ANOVA, Tukey’s multiple comparisons test).

Interestingly, the chemical properties of WS12, including a high log P = 5.052 ± 0.237 (calculated using Advanced Chemistry Development (ACD/Labs) Software V11.02 (1994–2018 ACD/Labs), allowed its lipid encapsulation in LNC600 composed of an oily core, including a mixture of triglycerides with medium chain fatty acids (caprylic and capric), that was surrounded by a lecithin crown and a hydrophilic surfactant (HS-PEG) (Fig. 2A). LNC600 containing WS12 were obtained using a phase inversion method (oil/water) due to different changes in temperature cycles. LNC600 obtained by this process were characterized according to their size and surface charge (Fig. 2B).

An LNC size characterization showed that empty LNC600 (25.2 ± 0.1 nm) and WS12-loaded LNC600 (24.1 ± 0.8 nm, LNC600-WS12) had the same diameter (Fig. 2B). Concerning surface charges, the zeta potential was not different between empty LNC600 (−7.0 ± 0.7 mV) and LNC600-WS12 (−5.1 ± 0.8 mV), as shown in Table 1.

**Table 1:** Characterization of empty and WS12-loaded lipid nanocapsules mean ± SD, n = 3.

	Mean diameter (nm)	PI	Zeta potential (mV)	EE of WS12 (%)
Empty LNC600	25.2 ± 0.1	0.163 ± 0.017	(−) 7.0 ± 0.7	—
LNC600-WS12 (1%)	24.1 ± 0.8	0.183 ± 0.007	(−) 5.1 ± 0.8	99

PI = LNC600 Polydispersity Index; EE = Encapsulation Efficiency.

Following the production and characterization of LNC600-WS12, we tested whether WS12 encapsulation affected its activation properties with regard for the TRPM8 channel. In these experiments, we performed calcium imaging on PC3 and PC3 TRPM8-overexpressing cells (PC3-M8) that were treated with empty or WS12-loaded LNC600 (Fig. 2C). These experiments showed that treatment with LNC600-WS12 or free WS12 (both at 10 nM) had similar effects on the activation of TRPM8 (Fig. 2Ci,iii). Notably, at lower agonist concentrations (1 nM), LNC600-WS12 induced a higher level of TRPM8 activation (increased activation by 41.2 ± 3.8%) than was observed for free WS12 (Fig. 2Cii,iii). Interestingly, exposing the cells to empty LNC600 had no effect on TRPM8 activation (Fig. 2Ci,ii). These results indicate that encapsulating WS12 potentiated the effect of this agonist on TRPM8 activity.

### Dynamics of LNC600-WS12 cellular uptake

**Figure 3:**
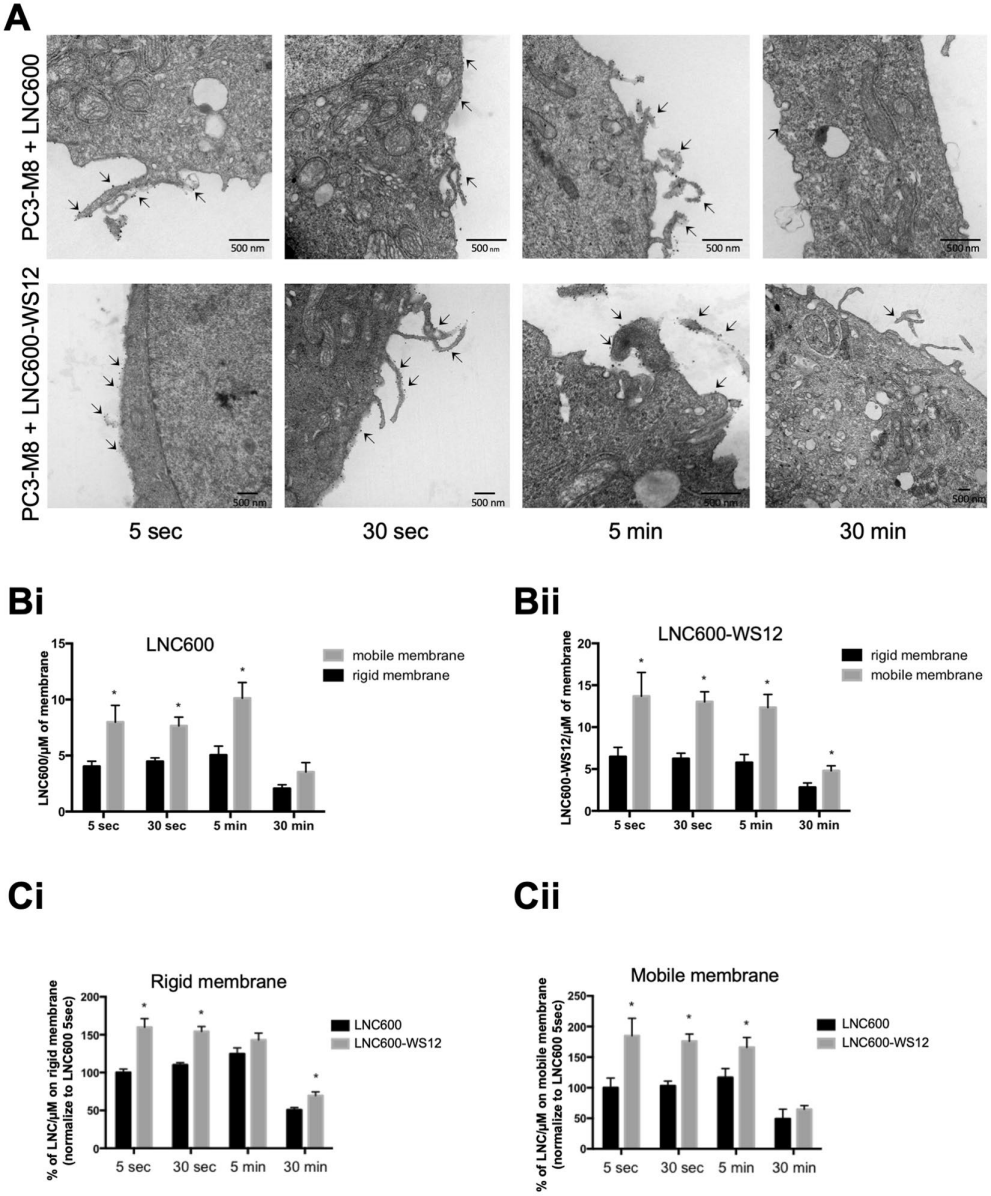
Plasma membrane penetration and dynamic localization of lipid nanocapsules. (**A**) Electron micrographs of PC3-M8 cells treated with 100 nM of empty LNC600 (upper panels) or 100 nM of LNC600-WS12 (lower panels) for different times (5 s, 30 s, 5 min and 30 min). Lipid Nanoparticles appeared as electron-dense spots located at the plasma membrane and are indicated by an arrow. (**B**) Bar graphs show the quantification of the data for empty LNC600 (**Bi**) or LNC600-WS12 (**Bii**) as µm of mobile or rigid plasma membrane at different timepoints after treatment with 100 nM of empty LNC600 or LNC600-WS12. (**C**) Bar plot showing the quantification of empty LNC600 or LNC600-WS12 in the rigid (**Ci**) and mobile (**Cii**) fractions of plasma membranes obtained from PC3-M8 cells at different timepoints after treatment with 100 nM of empty LNC600 or LNC600-WS12. *P < 0.05 (ANOVA, Tukey’s multiple comparisons test).

As mentioned above, encapsulating WS12 potentiates its effect on TRPM8 activation, allowing activation at a lower concentration than that achieved by free WS12. We next sought to determine how LNC600 act on cells to activate TRPM8 channels. To answer this question, we first studied the cellular localization of LNC600 and TRPM8 channels by electron microscopy in PC3-M8 cells at 5 s, 30 s, 5 min and 30 min after the cells were treated with 10 nM empty LNC600 or LNC600-WS12 (Fig. 3). For each treatment and at each time point, both empty LNC600 and LNC600-WS12 appeared as electron-dense spots located at the plasma membrane. Interestingly, on electron micrographs, the less deformable membranes (defined as rigid membrane) on which LNC600 localized could be distinguished from the more mobile membranes (pseudopodia, filopodia or invadopodia). Quantification of these electron dense spots revealed that both empty LNC600 (Fig. 3Bi) and LNC600-WS12 (Fig. 3Bii) were primarily located on mobile plasma membranes rather than rigid membranes regardless of the application duration, and the electron densities of spots on the plasma membranes dramatically decreased after 30 min, implying that they had been internalized.

Interestingly, LNC600-WS12 exhibited a higher rate of plasma membrane targeting than that observed for empty LNC600 (Fig. 3C). Indeed, compared to the empty LNC600, LNC600-WS12 targeted 60 ± 11.2% more of the rigid fraction (Fig. 3Ci) and 84.8 ± 28.35% more of the mobile fraction (Fig. 3Cii) of plasma membranes after 5 s. We also noticed a similar increase in the rigid fraction (an increase of  44.0 ± 6.5%) and the mobile fraction (an increase of 70.73 ± 11.88%) of plasma membranes after 30 s. In addition, in comparison to empty LNC600, LNC600-WS12 were clearly increased in the rigid fraction of plasma membranes after 30 min of treatment (an increase of 38 ± 5%) (Fig. 3Ci) and in the mobile fraction after 5 min (42.33 ± 16%) (Fig. 3Cii).

Furthermore, analysis of the fatty acid composition of PC3 and PC3-M8 cells treated with empty or WS12-loaded LNC600 for 0, 10 and 30 min using one-dimensional High-Performance Thin-Layer Chromatography (HPTLC) did not indicate any apparent modification of neutral lipid content. Table 2 shows that there were no significant differences in any of the evaluated classes of lipids (cholesterol, free fatty acid, triglyceride and esterified cholesterol) among untreated PC3 and PC3-M8 cells and cells treated with nanocapsules, indicating that treatment with LNC600 did not change the composition of the lipid membrane.

**Table 2:** Neutral lipid content of intracellular lipid vacuole: cholesterol (CHOH), free fatty acid (fFA), triglyceride (TG) and esterified CHOH.

	% CHOH/NL	% fFA/NL	% TG/NL	% Esterified CHOH/NL	% Total
**PC3**					
CTRL	40.04 ± 2.11	22.17 ± 4.62	10.68 ± 0.71	27.11 ± 2.68	100.00
LNC600 10 min	41.50 ± 0.98	18.59 ± 2.33	9.88 ± 0.37	30.03 ± 1.35	100.00
LNC600 30 min	42.74 ± 0.26	19.42 ± 0.41	10.72 ± 0.85	27.12 ± 1.24	100.00
LNC600-WS12 10 min	38.09 ± 4.04	22.17 ± 2.30	10.94 ± 0.67	28.81 ± 2.31	100.00
LNC600-WS12 30 min	34.77 ± 6.24	25.08 ± 2.60	11.05 ± 1.20	29.10 ± 3.01	100.00
**PC3-M8**					
CTRL	37.16 ± 1.18	21.36 ± 1.61	14.86 ± 0.60	26.63 ± 1.39	100.00
LNC600 10 min	39.33 ± 2.09	20.61 ± 2.63	13.29 ± 1.27	26.78 ± 3.26	100.00
LNC 30 min	38.45 ± 2.13	15.75 ± 4.61	15.81 ± 1.37	29.99 ± 3.85	100.00
LNC600-WS12 10 min	38.38 ± 3.70	16.68 ± 6.23	14.87 ± 1.63	30.07 ± 2.98	100.00
LNC600-WS12 30 min	32.18 ± 3.35	20.61 ± 3.05	20.01 ± 4.59	27.19 ± 2.83	100.00

Results are normalized to total neutral lipid (NL) levels in PC3 and PC3-M8 cells. 10^7^ cells were treated with 10 nM of empty LNC600 or LNC600-WS12 for 5 or 30 min. After total lipid extraction, total lipids were separated by HPTLC. Standards were used to quantify the different lipids. Lipid levels are expressed as the percentage of the total identified neutral lipid weight in the sample (mean values ± SEM). N = 3 independent analyses for each condition.

Electron microscopy data revealed that LNC600 were initially preferentially localized in the plasma membrane fraction and then later internalized (after 30 min). Thus, we next studied whether LNC600-WS12 induces rapid activation of TRPM8 channels by acting at the plasma membrane.

**Figure 4:**
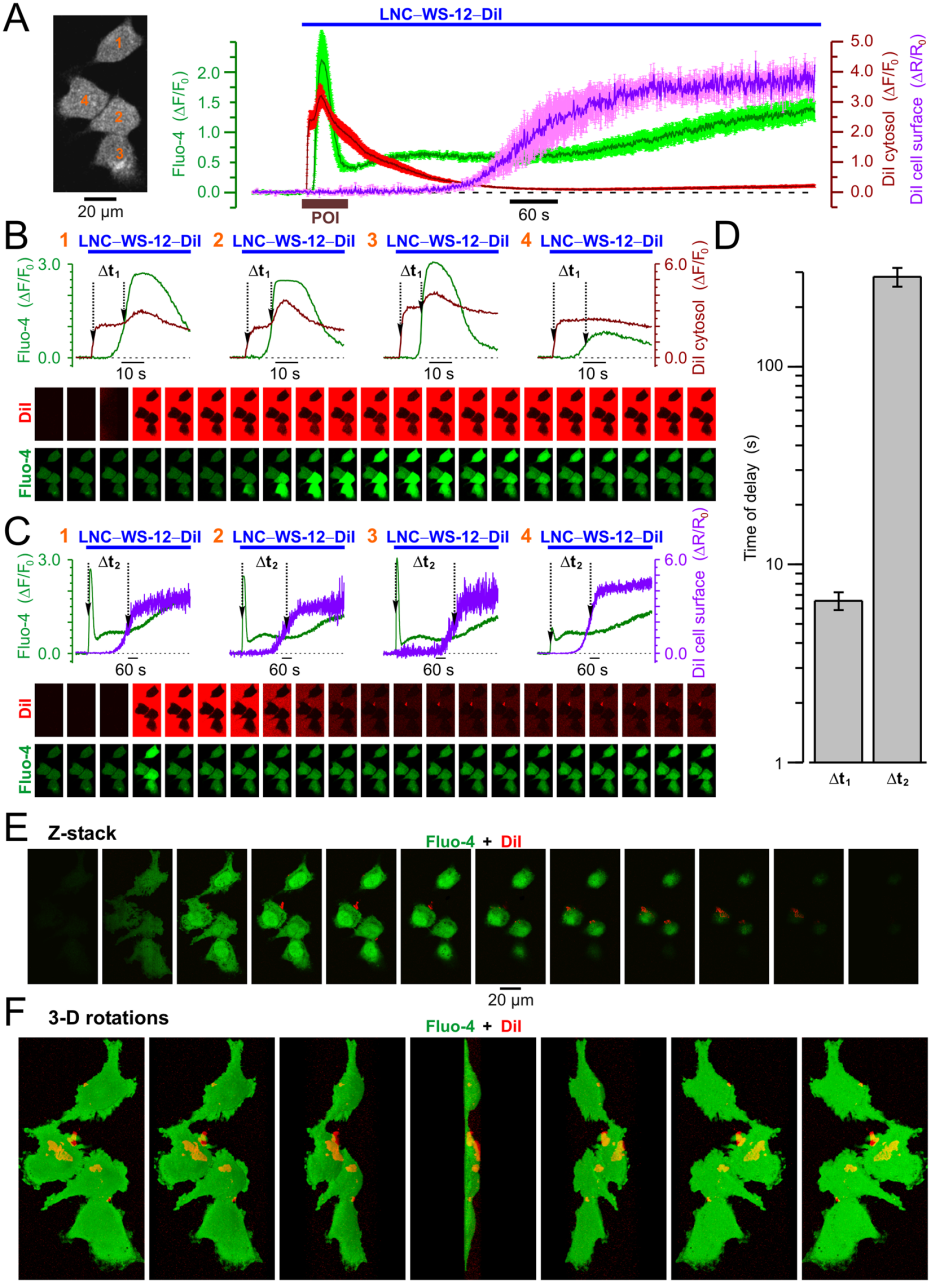
Confocal visualization of DiI/WS12-containing lipid nanocapsules (LNC600-WS12-DiI) and concurrent LNC-induced [Ca^2±^]_i_ responses in PC3 cell expressing stably TRPM8. (**A**) Plot (right) compares the dynamics of relative changes in Fluo-4 fluorescence reporting WS12-induced changes in intracellular Ca^2+^ concentration, [Ca^2+^]_i_ (ΔF/F0, green trace), DiI fluorescence reporting re-distribution of LNC content within the cell (ΔF/F0, red trace) and DiI fluorescence reporting aggregation of LNCs on the cell surface (ΔR/Ro, violet trace). The traces show mean ± S.E.M. signals from 4 cells (image, left). (**B**) Plots of Fluo-4 (green traces) and intracellular DiI (red traces) responses in 4 cells (numbered in A, left) during the period of interest (POI, brown bar in A, right) highlight the delay (Δt1) between internalization of LNC content and elevation of [Ca^2+^]_i_, respectively. (**C**) The same as B but the delay (Δt2) between the elevation of [Ca^2+^]_i_ (green traces) and aggregation of LNCs on the cell surface (violet traces) is highlighted. The galleries below the plots show every 8^th^ (**B**) and 100^th^ (**C**) image captured during the periods corresponding to the plots. (**D**) Plot displays mean ± S.E.M. values of Δt1 and Δt2 measured in 40 cells upon 17 independent experiments. Note that the LNC is internalized before [Ca^2+^]_i_ response initiation, while aggregation of LNCs on the cell surface became prominent >3 min later. (**E**,**F**) Visualization of the 3-dimensional (3-D) distributions of DiI and Fluo-4 fluorescence 20 min after stimulation of the cell with LNCs. The results are presented by galleries showing (**E**) every 2^nd^ x-y image obtained during Z-sectioning protocol (Z-stack) and (**F**) rotations of the reconstructed 3-D image around Y axis with 30° step (3-D rotations). Note LNC aggregates (red) on the cell surface.

**Figure 5:**
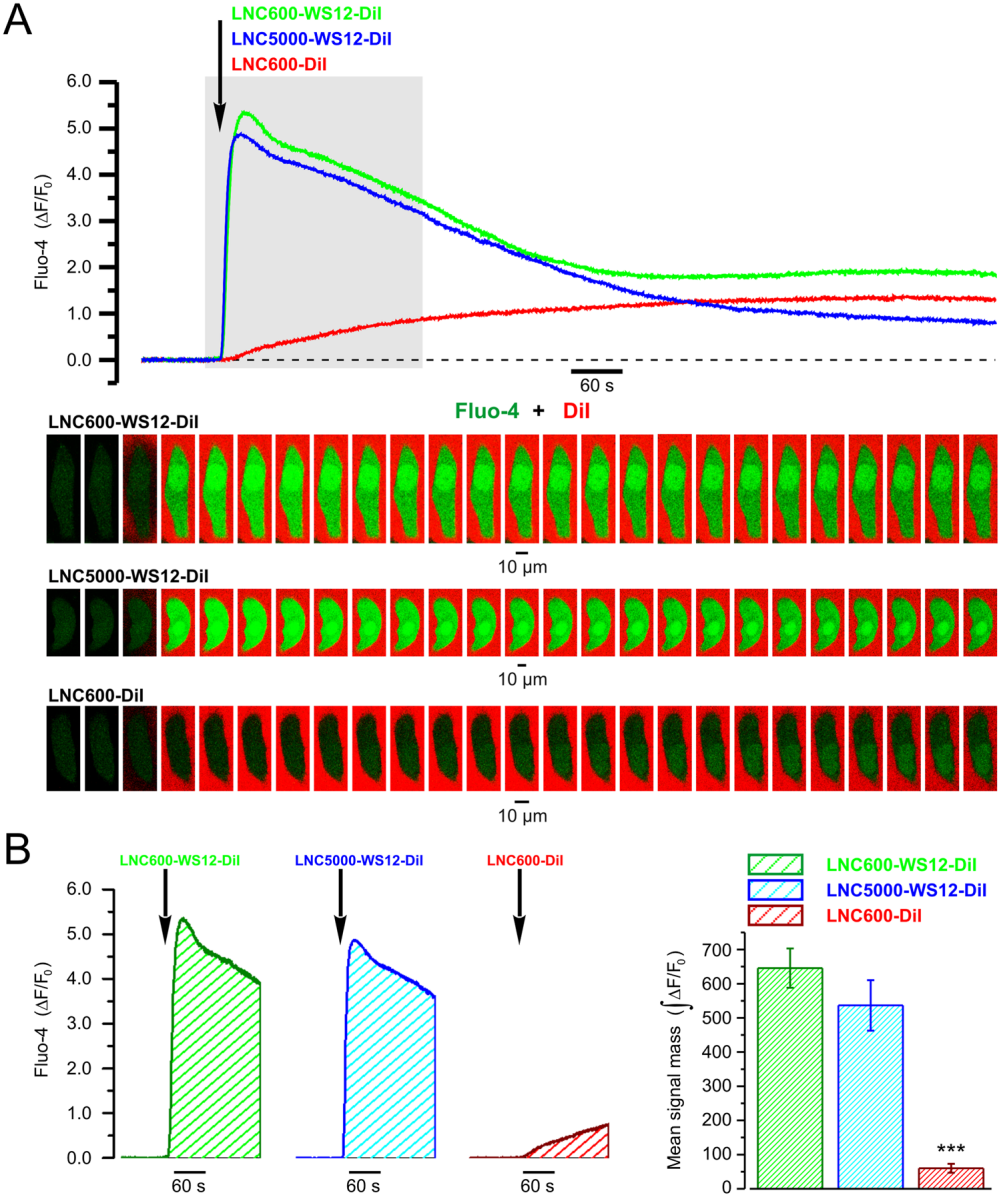
LNC600 and LNC5000 are both capable of delivering WS12 to the agonist binding site on TRPM8. (**A**) Plot (top) relates the dynamics of relative changes (ΔF/F_0_) in fluo-4 fluorescence induced by local application of DiI/WS12-containing LNC600 with PEG tails of 600 MW or 5000 MW (LNC600-WS12-DiI and LNC5000-WS12-DiI, respectively) and LNC600 containing DiI only (LNC600-DiI). The moment of LNC application of 300 times diluted LNC suspension is depicted by arrow. The traces and labels are shown in corresponding color. The gallery (bottom) shows every 50^th^ image of Fluo-4 (green) and DiI (red) fluorescence captured during the highlighted (top) period. The x-y time series confocal (optical slice < 0.8 µm) imaging (at 5 Hz) reported changes in [Ca^2+^]_i_ (Fluo-4) and presence of LNC600 in the vicinity of the cell (DiI). Note that unlike LNC600-WS12-DiI or LNC5000-WS12-DiI, LNC600-DiI failed to induce [Ca^2+^]_i_ transient, thus confirming TRPM8-specificity of the response. (**B**) The bar diagram plot (right) compares masses of Fluo-4 signal (left: examples; $\int ({\rm{\Delta }}F/{F}_{0})$) during first 180 s of application of LNC600-WS12-DiI (*n* = 6), LNC5000-WS12-DiI (*n* = 6) and LNC600-DiI (*n* = 8). ***P < 0.001, two-tailed Student’s *t*-test.

To relate the kinetics of cytosolic Ca^2+^ response caused by TRPM8 activation to the spatio-temporal pattern of LNC distribution, we conducted fast (acquisition rate 2.5–10 Hz) x-y confocal Ca^2+^ imaging on PC3 cells loaded with Fluo-4. The cells were stimulated by application of LNC600 encapsulating TRPM8 agonist WS12 and labeled with fluorophore DiI (LNC600-WS12-DiI). An example of the results obtained from single PC3 cell is presented in Fig. 4A. This approach revealed that DiI fluorescence started to rise in intracellular space on average 6.55 ± 0.68 s (*n* = 40) before the initiation of Ca^2+^ response reported by Fluo-4 fluorescence. This observation indicates that LNC600 may act as trans-plasma membrane cargo delivering their content (i.e. DiI) to the cell interior. The aggregation of LNC600 on the cell surface, however, became prominent on average 284.5 ± 30.7 s after the Ca^2+^ response initiation. Examples of distribution of the LNC600 aggregates on the surface of PC3 cells revealed with confocal Z-sectioning are shown in Fig. 4 and Supplemental Figs 1 and 2). Thus, confocal imaging of the responses induced by LNC600 with the fixed polyethylene glycol chain (PEG) length (i.e. 600 MW) did not allow us to elucidate unequivocally the site of their action. It was reported, however, that longer PEG decreases cellular uptake of LNC^26^. Bearing this in mind, we compared Ca^2+^ responses of PC3 cells to application of WS12-encapsulating LNC600 with two different lengths of PEG tail: 5000 MW (LNC5000-WS12-DiI) and 600 MW (LNC600-WS12-DiI). The DiI-labeled LNC600 with PEG of 600 MW but not encapsulating WS12 (LNC600-DiI) were used as control. This revealed that the extent of TRPM8 activation does not depend on the PEG length (Fig. 5), which suggests that WS12-encapsulating LNC600 act on the extracellular channel domain.

### LNC600-WS12 potentiates TRPM8-mediated cell migration *in vitro* and *in vivo*

**Figure 6:**
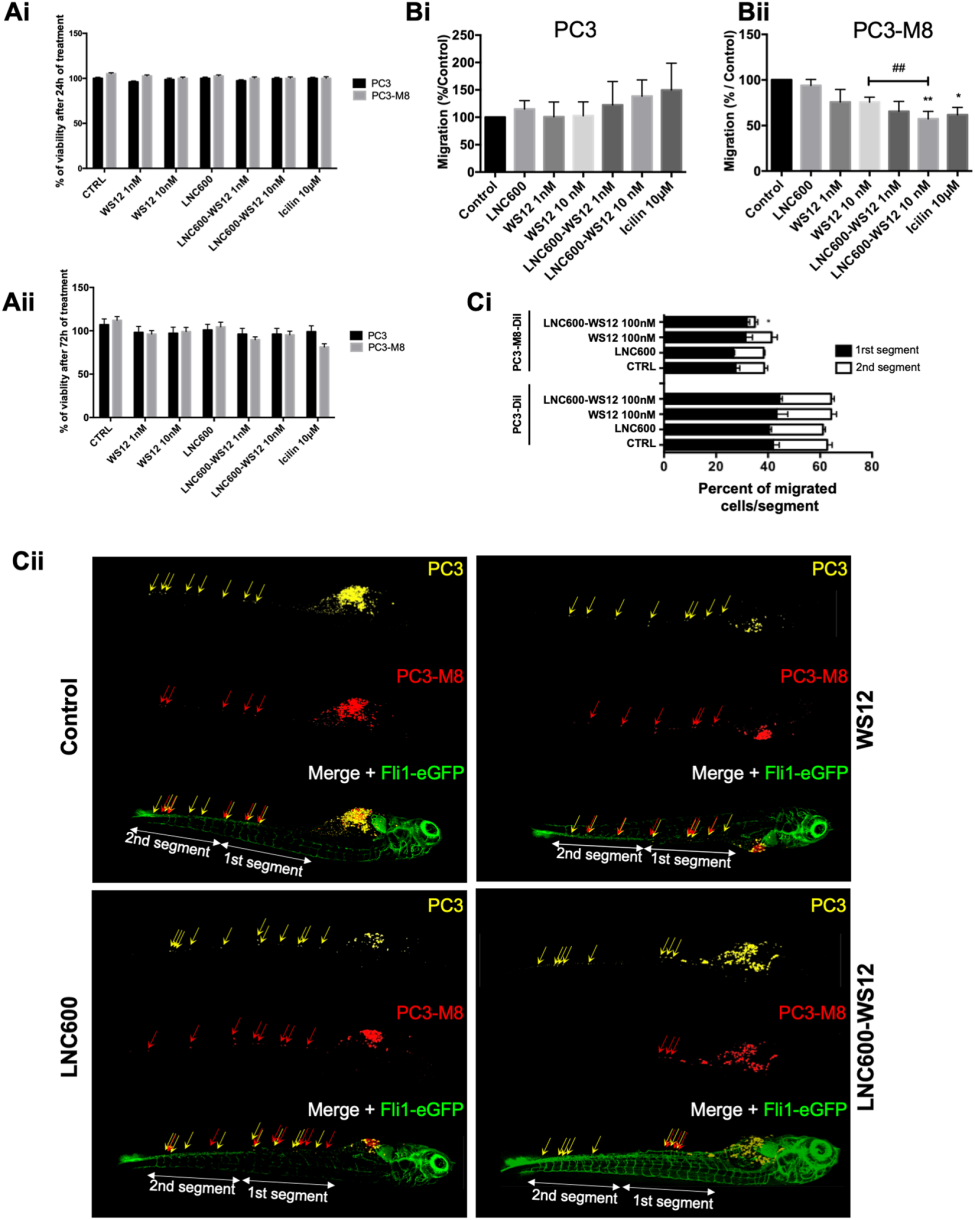
*In vitro* and *in vivo* effects of lipid nanocapsules on PC3-M8 cell viability and migration. (**A**) Cell viability assays performed with different TRPM8 agonists (WS12, empty LNC600, LNC600-WS12 or icilin) in PC3 and PC3-M8 cells and evaluated after 24 h (**Ai**) or 72 h (**Aii**) of incubation. MTS reagent was used to determine cell viability percentages (mean ± SEM; normalized to the control (CTRL) condition). (**B**) Transwell migration assays showing the effects of TRPM8 agonists on PC3 (**Bi**) or PC3-M8 (**Bii**) cells. The results are represented as a percentage of migrated cells normalized to the results observed under the CTRL condition (N = 9, mean ± SEM; *P < 0.05; **P < 0.01, ANOVA, Tukey’s multiple comparisons test for control and pairwise t-test comparison against 10 nM WS12 encapsulated or not condition, ^##^P < 0.01). (**C**) *In vivo* migration assays performed in zebrafish embryos. (**Ci**) Graph bar representing PC3 and PC3-M8 cell migration along the zebrafish tail following the application of 100 nM of empty LNC600, 100 nM of free WS12 or 100 nM of LNC600-WS12. For each condition, PC3 and PC3-M8 cells were counted and the results normalized to the total number of migrating cells (N = 15, mean ± SEM; *P < 0.05, ANOVA, Tukey’s multiple comparisons test). (**Cii**) Representative confocal images of Tg[Fli1-eGFP] zebrafish embryos (vasculature shown in green) injected with DiD-labeled PC3 (yellow) and DiI-labeled PC3-M8 (red) cells. Cell migration was quantified in two segments of the ventral part of the fish (1^st^ and 2^nd^ segments).

We next evaluated the toxicity of LNC600-WS12 by analyzing cell viability using MTS assays in PC3 and PC3-M8 cells (Fig. 6A). Neither empty LNC600 nor LNC600-WS12 affected cell viability in PC3 and PC3-M8 cells after 24 h (Fig. 6i) and 72 h (Fig. 6Aii) of treatment at 1 and 10 nM. Under otherwise identical conditions, free WS12 added at the same concentrations (1 and 10 nM) also had no toxic effect on cell viability.

However, it was recently shown that the migration of prostate cancer cells was inhibited when TRPM8 was activated by icilin and menthol^3–5,27^. We therefore tested whether the higher affinity agonist WS12 would inhibit cell migration and whether encapsulating the drug would affect this inhibitory activity. Transwell migration assays revealed that treatment with LNC600-WS12 significantly decreased migration at both concentrations (by 34.42 ± 11.09% at 1 nM and by 42.77 ± 8.253% at 10 nM, Fig. 6Bii). WS12 had an effect equivalent to that of icilin, and importantly, WS12 encapsulation increased how efficiency the agonist inhibited TRPM8-mediated cell migration (Fig. 6Bii). Indeed, treatment with LNC600-WS12 induced a 24.56% higher decrease than that induced by free WS12 at 10 nM, confirming our hypothesis that encapsulation of WS12 allows the use of lower agonist concentrations to activate TRPM8, as shown by calcium imaging (Fig. 2Ci,ii). Empty LNC600 had no effect on PC3 (Fig. 6Bi) or PC3-TRPM8 (Fig. 6Bii) cell migration.

After we accomplished *in vitro* confirmation that prostate cancer cell migration is inhibited by LNC600-WS12, we next investigated the significance of these effects *in vivo* using a xenograft assay in zebrafish embryos. We first tested the uptake of LNC600 dissolved in culture medium by zebrafish. For these experiments, we used the Tg(fli1:eGFP) zebrafish line, which allows the visualization of the vasculature (green)^28^. Confocal 3-D imaging revealed that LNC was taken up from culture water and accumulated in the intestinal tract of the zebrafish when they are treated with 1 µM or 100 nM WS12-loaded LNC600, but not those treated with 10 nM loaded LNC600 (Supplemental Fig. 3). We next injected a mix of PC3 cells labeled with DiD (yellow) and PC3-M8 cells labeled with DiI (red) into the yolk sacs of 2-day-old zebrafish and then treated the zebrafish water with empty 100 nM LNC600, LNC600-WS12 or free WS12 for 4 days. The zebrafish were subsequently fixed and subjected to confocal microscopy to track the migration of each cell population along the zebrafish tail (Fig. 6Ci). Cell migration was quantified by cell counting along the zebrafish tail, in which we evaluated 2 equal segments in the ventral tail (1^st^ and 2^nd^ segments, Fig. 6Ci). Interestingly, PC3-M8 cells migrated across 35.50 ± 3.07% shorter distances than were migrated by PC3 cells, and treatment with LNC600-WS12 further reduced PC3-M8 migration into the 2^nd^ tail segment of the zebrafish (77.83 ± 1.1%), but did not affect the total percent of PC3 M8 cells (Fig. 6Cii). At the same time, we found that treatment with free WS12 did not significantly modify PC3 and PC3-M8 migration, similar to what was observed for treatment with empty LNC600 (Fig. 6Cii). Overall, these results demonstrate that encapsulating WS12 into LNC600 potentiated the effect of WS12 on TRPM8 activation regarding prostate cancer cell migration and was not toxic *in vivo*.

## Discussion

The purpose of this study was to target cancer cell migration by acting on TRPM8 activity. Functional analyses of TRPM8 activity indicated that WS12 is the most sensitive and selective agonist for this channel, which we encapsulated and demonstrated that this encapsulation allowed 10 times lower concentration of the agonist in order to achieve similar activation of the TRPM8 channel. We used confocal and electron microscopy to demonstrate that the nanocarrier targeted the channel at the plasma membrane and to further characterize its effects on cell migration *in vitro* and *in vivo*. Interestingly, the encapsulated WS12 potentiated TRPM8-mediated prostate cancer cell migration in transwell assays as well as in the zebrafish model.

The TRPM8 channel is considered an important anti-cancer target due to its pattern of expression during prostate cancer carcinogenesis^29^; in particular, it is important because of the inhibitory role it plays against cell migration^2–5^. Currently, a variety of compounds are known to activate the TRPM8 channel and could be used as therapeutic agents to reduce cell migration in TRPM8-expressing cancers. In this study, we evaluated the three main agonists of TRPM8, including menthol, icilin and WS12, to find which would provide the most promising data when encapsulated based on the following criteria: the affinity and specificity of the agonist for channel activity. Here, we show that the concentrations of WS12 needed to induce equivalent levels of TRPM8 activation were at least 100 times lower that those required of menthol or icilin. These results are in accordance with previous studies revealing that the EC_50_ for menthol was 10.4 µM, which was 10 times higher than the EC_50_ for icilin (1.4 µM) and 100 times higher than that of WS12 (EC_50_ = 193 nM)^17^. These differences in affinity and sensitivity had not previously been shown for WS12, but some studies have suggested that WS12 acts on TRPM8 in a manner similar to that of menthol^17,30^. Indeed, when a mutation was made at glycine 805 in the putative S3 transmembrane domain of TRPM8 (this glycine is a critical amino acid for icilin sensitivity), the addition of WS12 induced TRPM8 activation at a level similar to that induced by menthol, but icilin did not induce TRPM8 activation^30^. The second criterion that we considered was the specificity of these compounds for the TRPM8 channel because different members of the TRP channel family can share the same modulators^31^. For instance, menthol has been shown to activate the TRPA1 channel^32,33^, the TRPV3 channel^34^ and to induce calcium release from the endoplasmic reticulum and Golgi *via* a TRPM8-independent pathway^35,36^. Similarly, icilin also activates TRPA1 channels^17,37^. Finally, with regard for the specificity of WS12 for TRPM8, no evidence has indicated that WS12 activates other TRP channels, including the TRPM3 and TRPV6 channels^17^. We believe that findings related to these two criteria, affinity and specificity for TRPM8, indicate that WS12 is the most efficient agonist of TRPM8 channel activity.

WS12, similar to most TRPM8 activators, is lipophilic and appears to be insoluble in water and consequently difficult to deliver *in vivo*. Therefore, we have proposed the use of LNC as a neutral carrier^11^ to improve the delivery and efficiency of this TRPM8 agonist. The LNC used in this study were composed of an oily liquid core that contained the compounds of interest that was surrounded by a layer of lecithins and hydrophilic surfactant (PEG of 600 MW), giving them a hybrid structure lying somewhere between that of polymeric nanocapsules and liposomes (a lipoprotein-like structure)^38^. Various types of anti-infection or anticancer drugs can be loaded at much higher rates in LNC than in liposomes. Furthermore, molecules conjugated to LNC can be integrated into the shell structure to enable active targeting. WS12 was encapsulated into LNC600, and the resulting compound, LNC600-WS12, exhibited increased affinity for TRPM8. The encapsulation of WS12 into LNC600 potentiated WS12-mediated TRPM8 activation by reducing the concentration of the drug required to activate TRPM8 by a factor of 10. WS12 is a hydrophobic compound, and its encapsulation into LNC600 seemed to improve its delivery to cells and limit WS12 precipitation and/or aggregation in solution. Its apparent aqueous solubility was increased by a factor of at least 60 (data not shown), and this could explain how its activity was potentiated by encapsulation. Encapsulation into LNC had previously been shown to potentiate the activities of other compounds, such as quercetin, a common plant-derived flavonoid, which had an apparent aqueous solubility that was increased by a factor of 100 when encapsulated in LNC^39^. Similarly, encapsulating hypericin, a non-specific kinase inhibitor, into LNC suppressed the aggregation of Hy in aqueous media, increased its apparent solubility and enhanced the production of singlet oxygen when results were compared to those obtained using the free drug^40^.

The potentiation of WS12 activity by encapsulation appeared to affect cellular targeting in addition to increasing the solubility of this lipophilic channel agonist. As a result of its composition, which included a heart oil, a crown phospholipid (Lecithin) and a non-ionic surfactant (PEG), LNC targets cells and, in particular, the lipid plasma membrane of cells and thereby functions like a non-selective trans-plasma membrane cargo delivery agent by penetrating the plasma membrane, allowing its contents to be internalized^41^. Indeed, we used electron microscopy (Fig. 3) to show that empty LNC600 and LNC600-WS12 target the plasma membranes of cells, resulting in a decrease in the number of LNC600 at 30 min after treatment. This observation suggests that LNC600 are internalized between 5 and 30 min after treatment. However, the fast activation of TRPM8 observed after LNC600-WS12 treatment (5 secs after LNC600-WS12 treatment) suggests that internalization is not necessary for WS12 to induce TRPM8 activation, and this effect could be explained by the fact that TRPM8 is localized on the plasma membrane. Because a single LNC could transport between 60 and 140 molecules of WS12, our hypothesis was that LNC600-WS12 released a higher concentration of WS12 on contact with the plasma membrane and that WS12 was then able to activate the TRPM8 channel. The plasma membrane activities of LNC600 are unclear and have not been explored in other studies that have supported the notion that LNC600 need to be internalized to be able to act^41^. Here, we further show that LNC have an extracellular action and their internalization is not required. Indeed, the LNC5000-WS12 induced the same calcium response concerning its kinetic and amplitude. LNC with longer PEGs at their surfaces have been previously shown to be less internalized by cancer cells due to the decrease of probability of LNC adsorption at the plasma membrane^26^. Paillard *et al*. reported that the internalization of LNC can happen quickly (after 2 min exposure) and that they can pass through *via* different endocytosis pathways, with the mode of transport being dependent on the size of the LNC; for example, the clathrin/caveolae-independent pathway can be used with a contribution of endogenous cholesterol. However, in this study, we demonstrate that the LNC600 were not incorporated into the plasma membrane because only insignificant changes were observed in the cells’ lipid composition (Table 2). A major concern when using nanocarriers *in vivo* is their potential to induce cytotoxic effects. LNC cytotoxicity has already been evaluated in different cell lines^41,42^, and they have been found to have no toxicity at low doses (less than 1 µM)^42^. Interestingly, we did not observe any cytotoxic effect when empty LNC600 or LNC600-WS12 were applied in *in vitro* viability assays or an *in vivo* zebrafish model, even though encapsulation of the agonist clearly targeted TRPM8-mediated migration. Indeed, the results of an *in vitro* Transwell assay (Fig. 6) confirmed findings reported in previous studies that revealed that the menthol- or icilin-mediated effects on TRPM8 expression and activation included the inhibition of PCa cell migration^3–5,43^. In this study, we further confirmed that WS12 also blocks PCa cell migration by inhibiting TRPM8. Most importantly, we validated the anti-migratory role of TRPM8 and its agonist WS12 *in vivo* using a xenograft zebrafish embryo model. Delivering WS12 in LNC600 resulted in the more efficient inhibition of TRPM8-mediated cell migration both *in vitro* and *in vivo*, confirming that LNC600 can deliver this TRPM8 agonist *in vivo*. Because migration is a key process during metastatic development, these results strengthen the hypothesis that TRPM8 exerts a protective role against prostate metastatic cancer progression. Furthermore, our results demonstrate that LNC600-WS12 can be used to target the TRPM8 channel and that encapsulation potentiated its protective role against prostate metastatic cancer progression. Furthermore, functionalization of LNC using an antibody against PSMA (Prostate Specific Membrane Antigen) can be used to specifically target the prostate and limit possible side effects of TRPM8 activation in other organs. The functionalization of nanocarriers containing an antibody against PSMA has already been tested, and the results of an *in vivo* biodistribution assay showed that nanocarrier accumulation increased in the prostate^9^. The biodistribution of empty LNC600 has previously been explored in mice^44^, and the results seem to support the promise of using LNC600-WS12 in further *in vivo* studies in mice and possibly in future therapeutic applications developed to prevent PCa cell migration. Another potential use of these nanotools is introducing a radioisotope, such as a fluorine atom (^18^F), which does not drastically alter WS12 activity, as an anti-cancer compound to target TRPM8^16^. Even though the addition of the fluorine atom decreases by 25% the efficiency of the channel agonist, it brings a radiodiagnostic and potentially radiotherapeutic properties since it could be for instance used in PET imaging. Such a radiotherapeutic approach of targeting prostate cancer cells expressing TRPM8 would be more accurate than surgery and decrease at the same time the associated side effects such including impotency and incontinence.

## Conclusion

Our results demonstrate that encapsulating the TRPM8 agonist in LNC potentiated the inhibition of prostate cancer cell migration through the activation of the channel. These results provided proof-of-concept support for using TRPM8 channel-targeting nanotechnologies based on LNC to develop more effective methods for inhibiting prostate cancer cell migration *in vivo* and to explore the modulation of additional channels because several TRPs are implicated in different types of cancers^45,46^. Finally, the specific LNC nanotools described here could also be used for other diseases affected by TRPM8, which include migraine^47^, pain^48^ and male fertility^49^.

## Material and Methods

### Drugs and chemicals

Menthol (Sigma-Aldrich, France), was resuspended in ethanol to a final concentration of 10 mM and stored at −20 °C, according to the manufacturer’s instructions. Icilin (Tocris, France) was resuspended in DMSO to a stock concentration of 20 mM and stored at −20 °C. WS12 (Tocris, France), was resuspended in ethanol to a stock concentration of 1 mM and stored at −20 °C according to the manufacturer’s instructions. Drugs were diluted with the culture medium shortly before performing the assays.

### Cell culture and transfection

We used human Prostate Cancer cells (PC3) with stable TRPM8 overexpression (PC3-M8). PC3-M8 cells were obtained by a stable transfection of pcdna4-TRPM8 vector. PC3 and PC3-M8 cells contain a stable transfection of the luciferase gene and were grown in RPMI (Invitrogen Ltd, UK) supplemented with 10% of fetal bovine serum (Pan Biotech), L-glutamine (5 mM; Sigma-Aldrich, France) and PenStrep (100 mg/ml; Sigma-Aldrich, France).

### Calcium imaging

Cytosolic Ca^2+^ concentrations were measured using the ratiometric dye Fura-2/AM (Invitrogen Ltd, UK). Cells were loaded with 2.5 μM of Fura-2/AM (Interchim, France) for 45 min then washed and immersed in the extracellular solution containing 145 mM NaCl, 5 mM KCl, 2 mM CaCl_2_, 1 mM MgCl_2_, 10 mM N-(2-hydroxyethyl)-piperazine-N′-ethanesulfonic acid (HEPES), 10 mM glucose (NaOH to pH 7.35). Observations were performed at 37 °C on an Eclipse Ti microscope using an S Fluor 20×/0.75 NA objective lens (both from Nikon). Images were collected through a Rolera EM-C2 charge-coupled device (CCD) camera (QImaging) controlled with Metafluor software (Molecular Devices). Data were then analyzed with GraphPad Prism 6 software (GraphPad Corporation).

### Lipid nanocapsules formulation

Lipid nanocapsules were made of Labrafac Lipophile WL 1349 (caprylic/capric triglyceride), Phospholipon 90G (soybean lecithin at 97.1% of phosphatidylcholine), and Solutol HS15 (a mixture of free polyethylene glycol 660 and polyethylene glycol 660 hydroxystearate) generously provided by Gattefosse S.A.S. (Saint-Priest, France), Phospholipid GmbH (Köln, Germany), and Laserson (Etampes, France), respectively. Deionized water was obtained from a Milli-Q plus system (Millipore, Paris, France). WS12 from Tocris (Lille, France), DiI, other chemical reagents and solvents were obtained from Sigma-Aldrich (Saint-Quentin Fallavier, France) and used as received.

#### Preparation of blank, DiI and WS12-loaded LNC600

LNC600 were formulated at a nominal size of 25 nm in water using a phase inversion method of an oil/water system, as described by Heurtault *et al*.^11^. Briefly, the oil phase containing Labrafac (252 mg), Solutol (408 mg) and Phospholipon 90 G (37.5 mg) was mixed with the appropriate amounts of WS12 (1.4 mg), Milli-Q water (540 µL) and NaCl (44 mg), and heated under magnetic stirring up to 80 °C. The mixture was subjected to 2–3 temperature cycles from 40 to 80 °C under magnetic stirring. Then, it was cooled to 55 °C, 3.3 mL of cold Milli-Q water (0 °C) were added, and the suspension (4.2 mL) was stirred at room temperature for another 10 min before further use.

Fluorescent LNC600: DiI-loaded LNC600 were formulated similarly with 3% of DiI (1 mg/mL stock solution).

Blank LNC600 were prepared without any additional drug.

The different formulations were then purified using disposable PD-10 desalting columns (Sephadex G-25 for gel filtration as stationary phase, Amersham Biosciences). LNC suspensions were deposited on the column, and the purified LNC600 were collected in Milli-Q water (dilution factor of gel filtration = 4).

#### Drug loading

The drug loading of purified LNC suspensions was directly determined by reversed phase – high performance liquid chromatography (RP-HPLC). RP-HPLC analyses were performed on a Shimadzu LC2010-HT (Shimadzu, Tokyo, Japan). A 5 µm C_4_ QS Uptisphere 300 Å, 250 × 4.6 mm column (Interchim, France) was used as the analytical column. The column was heated to 40 °C. The mobile phase consists of a mixture of eluent A (trifluoroacetic acid 0.1% in H_2_O) and eluent B (trifluoroacetic acid 0.085% in CH_3_CN) at a flow rate of 1 ml/min. The linear gradient was 0% to 80% of eluent B in 35 min and detection was performed at 254 nm. A 5 mM stock solution of WS12 was prepared in DMSO for the calibration curve. Concentrations of 10–250 µM of WS12 in DMSO were prepared from this stock. Each sample was injected (40 µL) into the RP-HPLC column. A calibration curve (*y* = 31810*x*, *r*^*2*^ = 0.9999) was obtained by linear regression of drug concentration (*x*, µM) versus the peak area (*y*).

#### Characterizations

Size and zeta-potential measurements were performed using a Zetasizer Nano-ZS (Malvern Instruments Inc. Worcestershire, UK). Formulations were diluted by 1/60 and measured in Milli-Q water.

### Analysis of neutral lipid content

PC3 and PC3-M8 cells (in 175 cm^2^ flasks) were pretreated with empty or WS12-loaded LNC during 10 or 30 min. Total lipids from cells were extracted according to Bligh and Dyer method^50^. Total lipids were separated by High Performance-Thin Layer Chromatography (one-dimensional HPTLC) and dedicated silica gel HPTLC plates (10 × 20 cm, Merck Millipore). After staining, samples and reference standards for cholesterol (CHOH), Free Fatty Acid (fFA), Triglyceride (TG) and esterified cholesterol (esterified CHOH) (Sigma-Aldrich, France) were visualized by brown coloration after carbonization using the TLC-Visualiser (Camag). Visual inspection of plates was performed using a Reprostar 3 (Camag) and white light, before and after derivatization. Densitometric analysis was performed using the winCATS software (Camag). Precisely, samples, in the form of 7-mm bands, were spotted at a constant application rate of 150 nL/s under nitrogen For development, the mobile phase moderately polar used Linear ascending development was carried out in 20 cm × 10 cm twin trough glass chamber (Camag Muttenz Switzerland) pre-equilibrated with the mobile phase. Optimal chamber saturation time for mobile phase was 15 min at room temperature. The length of development was 7 cm. 20 ml of mobile phase were used with 10 mL in the trough containing the plate and 10 mL in the other trough Subsequent to the development, HPTLC plates were dried under a ventilated hood for 90 min after migration. Dried plates were treated by immersion in acid solution for 2 min. After 2 hours of drying, plates were placed on a plate heater (TLC Heater 3, Camag Muttenz Switzerland) for 20 min at 160 °C to allow carbonization. Densitometric analysis was performed using a TLC/HPTLC densitometer (Camag model 76510 scanner, Reprostar III, Camag, Muttenz, Switzerland). Standards were used to quantify the different lipids. Lipids levels were expressed as the percentage of total identified neutral lipid weight in the sample.

### Microscopy

#### Transmission electron microscopy

Cells were fixed overnight in 4% paraformaldehyde + 2.5% glutaraldehyde prepared in PBS buffer and post-fixed in 1% osmium tetroxide in the same buffer for 15 min. After acetonitrile dehydration, the pellets were embedded in Epon. Serial thin sections (90 nm) were cut using a Leica EM UC7 ultramicrotome and collected on 150 mesh hexagonal barred copper grids. After staining with 2% uranyl acetate prepared in 50% ethanol and incubation with a lead citrate solution, sections were observed on a Hitachi H-600 transmission electron microscope. Electronic microscopy analysis was performed using a confocal microscope (LSM 700; Carl Zeiss, Inc.) and Fiji analysis software^51^.

#### Confocal imaging

Confocal imaging was performed with LSM 510 META confocal workstation using a Plan-Neofluar 40 × 1.3 NA objective (Carl Zeiss, Germany). The illumination intensity was attenuated to 1–7% (depending on the laser line) with an acousto-optical tunable filter (Zeiss, Oberkochen, Germany). To optimize signal quality, the pinhole was set to provide a confocal optical section < 0.8 µm. To avoid any bleed-through of the fluorescence signal in multi-dye experiments, imaging was performed using the line-by-line multitrack mode of the confocal scanner. The photomultiplier gain and offset were set individually to achieve similar signal intensity at each channel and remove sub-signal noise from the images. Fluo-4 was excited by the 488 nm line of 500 mW Argon ion laser (Laser-Fertigung, Hamburg, Germany) and the fluorescence was captured at wavelengths 505–545 nm. DiI was excited by the 543 nm line of 5 mW Helium/Neon ion laser and the fluorescence was captured at wavelengths above 560 nm. For cell stimulation, the suspension of LNC600 containing 100 µM WS12 and 3% (w/v) DiI was diluted 1000-fold and applied to the PC3 cells during the x-y time series confocal (optical slice < 0.8 µm) imaging. The x-y time series imaging was performed at 2.5–10 Hz depending on the size of the region of interest. Changes in Fluo-4 and DiI fluorescence intensities (ΔF) averaged within the cell were normalized to the corresponding mean values before the LNC600 application (F_0_). To unravel the dynamics of LNC aggregation on the cell surface, the intensity of DiI fluorescence at the plasma membrane region was divided by its intensity in the extracellular space (R = F_PM_/F_o_), and relative changes in this ratio following LNC application (ΔR/R_o_) were plotted over time. Confocal Z-sectioning (optical slice < 0.8 µm; Z-step < 0.4 µm) for visualization of the 3-dimensional distributions of DiI and Fluo-4 fluorescence was performed >20 min after the application of the LNC-containing solution. Image processing was carried out using LSM 5 software (Zeiss, Oberkochen, Germany) and with custom routines written in IDL (Research Systems, Inc., Boulder, CO, USA). Statistical analysis was performed using MicroCal Origin (MicroCal Software Inc., Northampton, MA, USA).

### Viability assay

The cell viability was assessed using standard MTS assay according to the manufacturer’s instructions (Promega, France). The protocol was performed as follows: PC3 or PC3-M8 cells (3000 cells per well) were cultured in a 96-well plate (Corning Incorporated, France). The cells were treated with two concentrations of WS12 (1 and 10 nM), LNC600-WS12 (1 and 10 nM), 10 nM of empty LNC, 10 µM of icilin or EtOH with 10 wells per group for statistical analysis, and then cultured for 24 h or 72 h; 20 μL of MTS solution (Promega, France) was added prior to drawing off the medium for luminescence absorbance (at 490 nm) measurement using plate-reader (Tristar LB-942, Berthold, France) after 3 h of incubation of the reagents in the dark at 37 °C.

### Migration assay

#### Transwell

Cells were first harvested from the culture dish and 5 × 10^4^ cells in 200 μL of 2% serum medium were transferred to the Transwell inserts (the top compartment, 8-μm pore size, Corning) and 1 mL of 10% serum medium was placed in the lower chamber. Following incubation at 37 °C for 22 h in a cell culture incubator, cells on the upper surface of the filters were removed with cotton swabs, filters were washed with PBS, fixed in methanol, and stained with crystal violet. Cells that had moved to the lower surface of the filter were counted under the microscope. Migrated cells in each field were quantified. Results are presented as relative migration by setting the migrating cell number of control cells as 100.

#### *In vivo* zebrafish

Zebrafish were handled in compliance with local care regulations according to the French and European Union guidelines for the handling of laboratory animals (Directive 2010/63/EU of the European Parliament and of the Council of 22 September 2010 on the protection of animals used for scientific purposes). The experimental procedures carried out on zebrafish were reviewed and approved by the local Ethics Committee from the Animal Care Facility of the University of Lille – Region Hauts-de-France (approval number 2018011722529804). Fish were kept at 28 °C in aquaria with day/night light cycles (10 h dark versus 14 h light periods). The developing embryos were kept in an incubator at constant temperatures. Four hours post-fertilization (hpf) embryos were incubated with 0.2 mM 1-phenyl-2-thio-urea (Sigma) to prevent pigmentation. At 1 day post-fertilization (dpf), zebrafish embryos were dechorionated by incubation in 1% pronase (Sigma) for 1 min. At 2 dpf zebrafish dechorinated embryos are anesthetized with 0.04 mg/mL of tricaine (MS-222, Sigma) and transferred onto an agarose gel for microinjection.

CM-DiI or CM-DiD (Molecular Probes) labeled cells (at 2.10^7^ cells/mL) are loaded into a pulled glass micropipette (15 µm internal- and 18 µm external-diameter) attached to an air driven FemtoJet microinjector (Eppendorf). Cells are injected in the yolk sac of zebrafish embryos using 100 ms pulse time and 300 hPa positive pressure. One hour after the injection, the embryos are examined for the presence of a fluorescent cell mass in the yolk sac and then transferred to an incubator at 30 °C. At one-day post-injection (dpi) injected zebrafish are checked for normal morphology and transferred to 32 °C. At 6 dpi, the injected zebrafish are fixed with 4% paraformaldehyde for confocal imaging.

WS12, LNC600-WS12, or empty LNC are added to fish water 1 hour after cell transplatation.

### Statistical analysis

Values are expressed as means ± SEM. The statistical significance of differences between groups was determined by analysis of variance (ANOVA) followed by pairwise comparison using Tukey’s method for calcium imaging recordings, quantification of the electron dense spots by electronic microscopy and for migration assays. Differences in means with a P < 0.05 were considered statistically significant. Statistical analyses were performed using GraphPad Prism 6 software (GraphPad Corporation).

## Supplementary information

Supplementary Figures 1-3

## Data Availability

No datasets were generated or analysed during the current study.
