# Supplementary material for: Encapsulation of a TRPM8 Agonist, WS12, in Lipid Nanocapsules Potentiates PC3 Prostate Cancer Cell Migration Inhibition through Channel Activation
Source: Sci Rep. 2019 May 28;9:7926. doi: 10.1038/s41598-019-44452-4 (PMC6538610; doi:10.1038/s41598-019-44452-4)
Supplement: Supplementary file 1 — Supplementary Figures 1-3 [file 41598_2019_44452_MOESM1_ESM.docx]

**Supplementary information**

**Encapsulation of a TRPM8 Agonist, WS12, in Lipid Nanocapsules Potentiates PC3 Prostate Cancer Cell Migration Inhibition through Channel Activation**

Grolez G.P^1*^, Hammadi M.^2*^, Barras A.^2^, Gordienko D.^1^, Slomianny C.^1^, Völkel P.^3^, Angrand PO.^3^, Pinault M.^4^, Guimaraes C.^4^, Potier-Cartereau M.^4^, Prevarskaya N.^1^, Boukherroub R.^2^ and Gkika D.^1^

*^1^* *Univ. Lille, Inserm, U1003 - PHYCEL - Physiologie Cellulaire, Laboratory of Excellence, Ion Channels Science and Therapeutics, F-59000 Lille, France, , Université de Lille, Villeneuve d'Ascq, France. ^2^ Univ. Lille, CNRS, Central Lille, ISEN, Univ. Valenciennes, UMR 8520, IEMN, F-59000 Lille, France. ^3^ Cell Plasticity & Cancer, Inserm U908/University of Lille, Lille, France; CNRS, Lille, France. ^4^ Université de Tours, Nutrition, Croissance et Cancer, Inserm UMR1069, Tours, France- Ion channel Network and Cancer-Canceropole Grand Ouest, (IC-CGO).* ^*^ Co-first author.

**
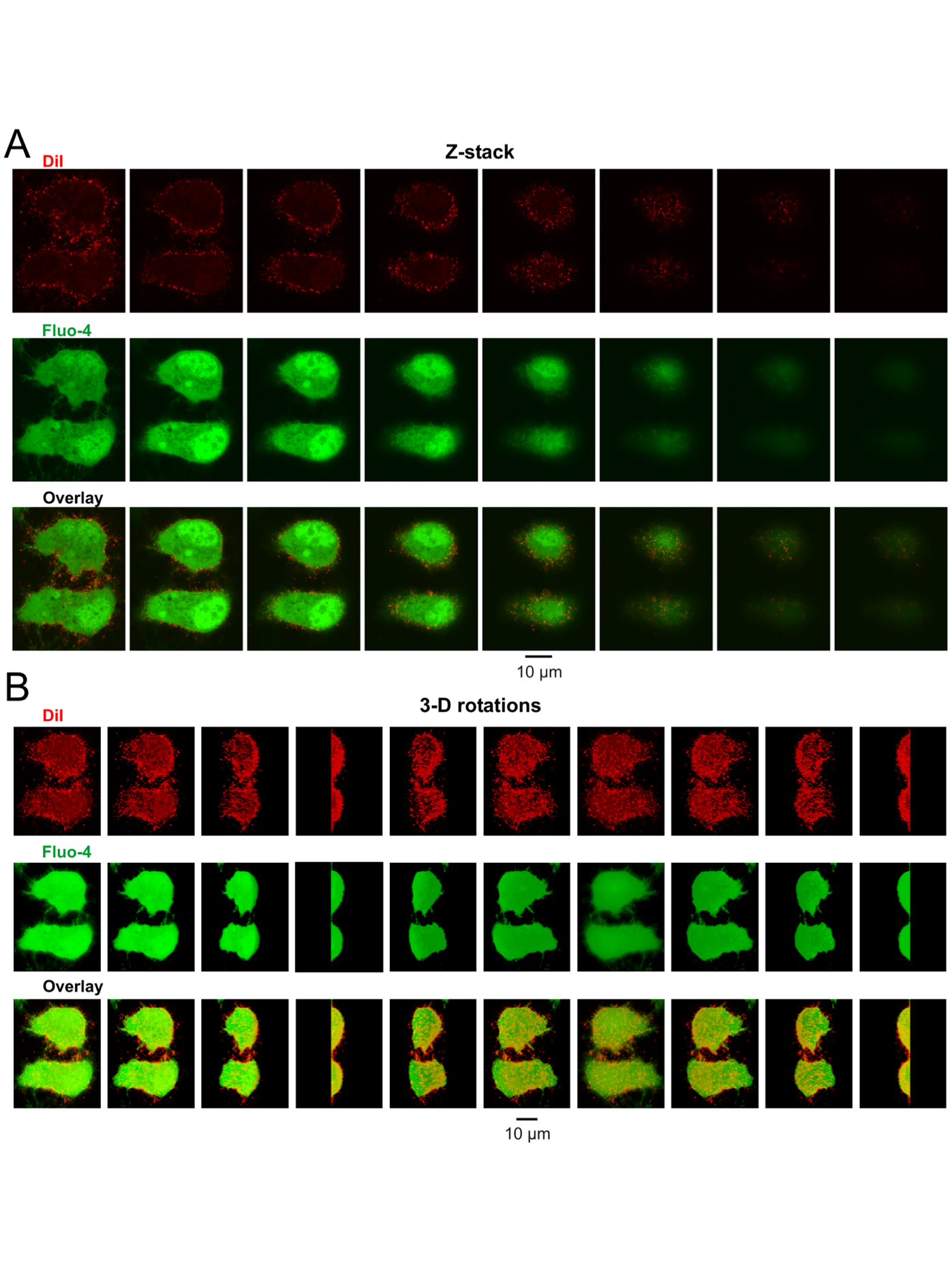
**

**Supplemental 1.** **Confocal analysis of lipid nanocapsule localization.**

Visualization of Z-stack **(A)** and 3-dimensional (3-D) **(B)** distributions of DiI and Fluo-4 fluorescence following stimulation of PC3 cells with LNC600-WS12-DiI. Confocal Z-sections (optical slice < 0.8 µm) were obtained > 20 min after the application of an LNC-containing solution. Z-sections are presented as galleries showing every 2^nd^ x-y image obtained during the Z-sectioning protocol (Z-stack), and rotations of the reconstructed 3-D images are shown around the Y axis with a 30º step (3-D rotations) for both Fluo-4 and DiI fluorescence and their overlay, as indicated.

**
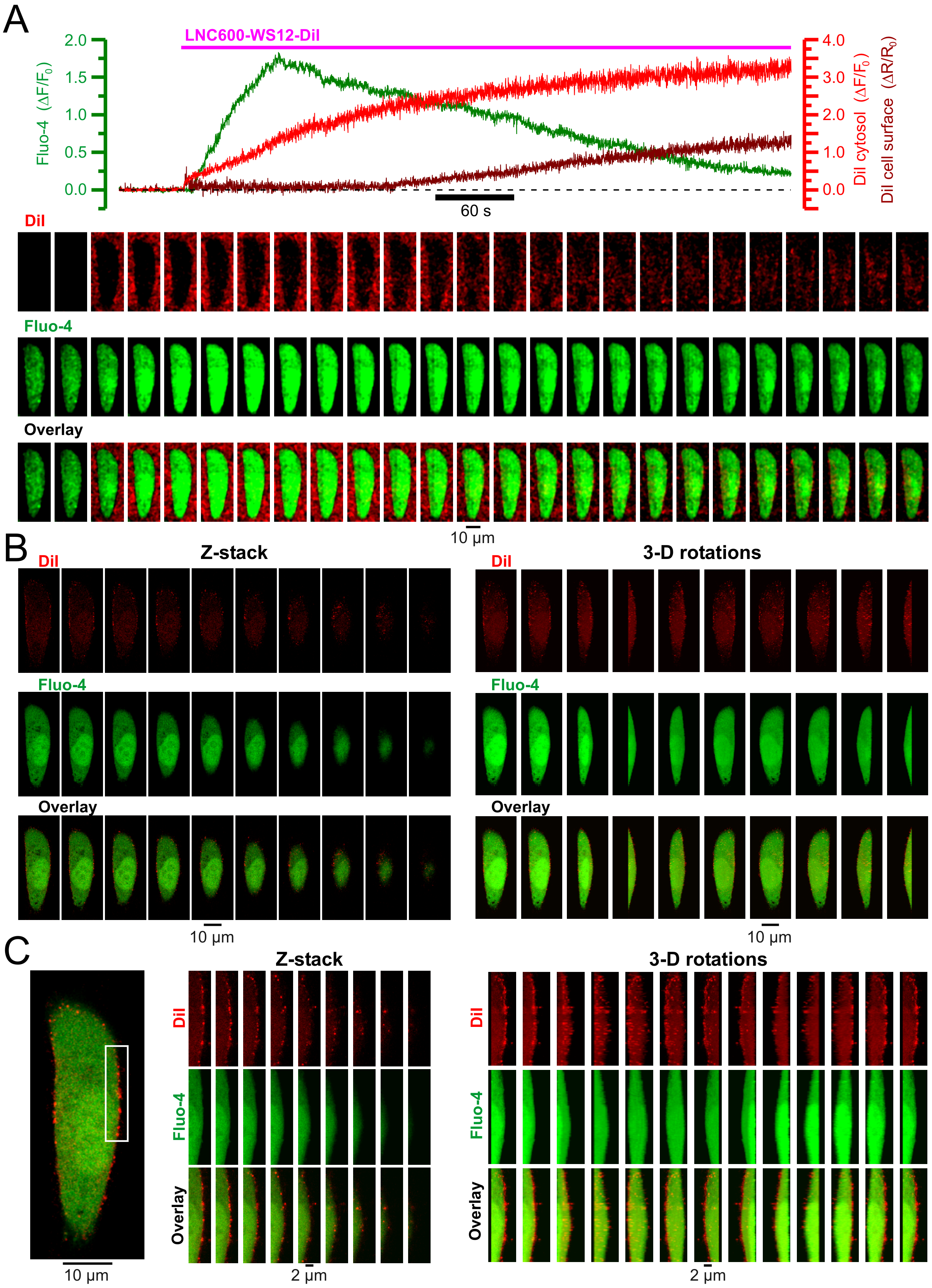
**

**Supplemental 2.** **Confocal visualization of DiI/WS12-containing lipid nanocapsules (LNC600-WS12-DiI) and concurrent LNC-induced [Ca^2±^]_i_ responses in PC3 cell constantly expressing TRPM8. (A)** Plot (top) compares the dynamics of relative changes in Fluo-4 fluorescence reporting WS12-induced changes in intracellular Ca^2+^ concentration, [Ca^2+^]_i_ (ΔF/F_0_ green trace), DiI fluorescence reporting re-distribution of LNC content within the cell (ΔF/F_0_ red trace) and DiI fluorescence reporting aggregation of LNC600 on the cell surface (ΔR/R_o_ wine trace). Note that the LNC content reached the intracellular space before [Ca^2+^]_i_ response initiation, while aggregation of LNC600 on the cell surface became prominent > 3 min later. The gallery (bottom) shows every 200^th^ captured image of Fluo-4 and DiI fluorescence, and their overlays, as indicated. **(B-C)** Visualization of the 3-dimensional (3-D) distributions of DiI and Fluo-4 fluorescence 20 min after stimulation of the cell with LNC600. The results of whole-cell **(B)** and ROI (**C**; left: ROI is outlined on the whole-cell image) Z-sectioning are presented by: (middle) galleries showing every 2^nd^ x-y image obtained during Z-sectioning protocol (Z-stack) and (right) rotations of the reconstructed 3-D image around Y axis with 30º step (3-D rotations) for Fluo-4 and DiI fluorescence, and their overlay, as indicated. Note LNC conglomerates on the cell surface and homogeneous DiI signal from the cell interior.

**
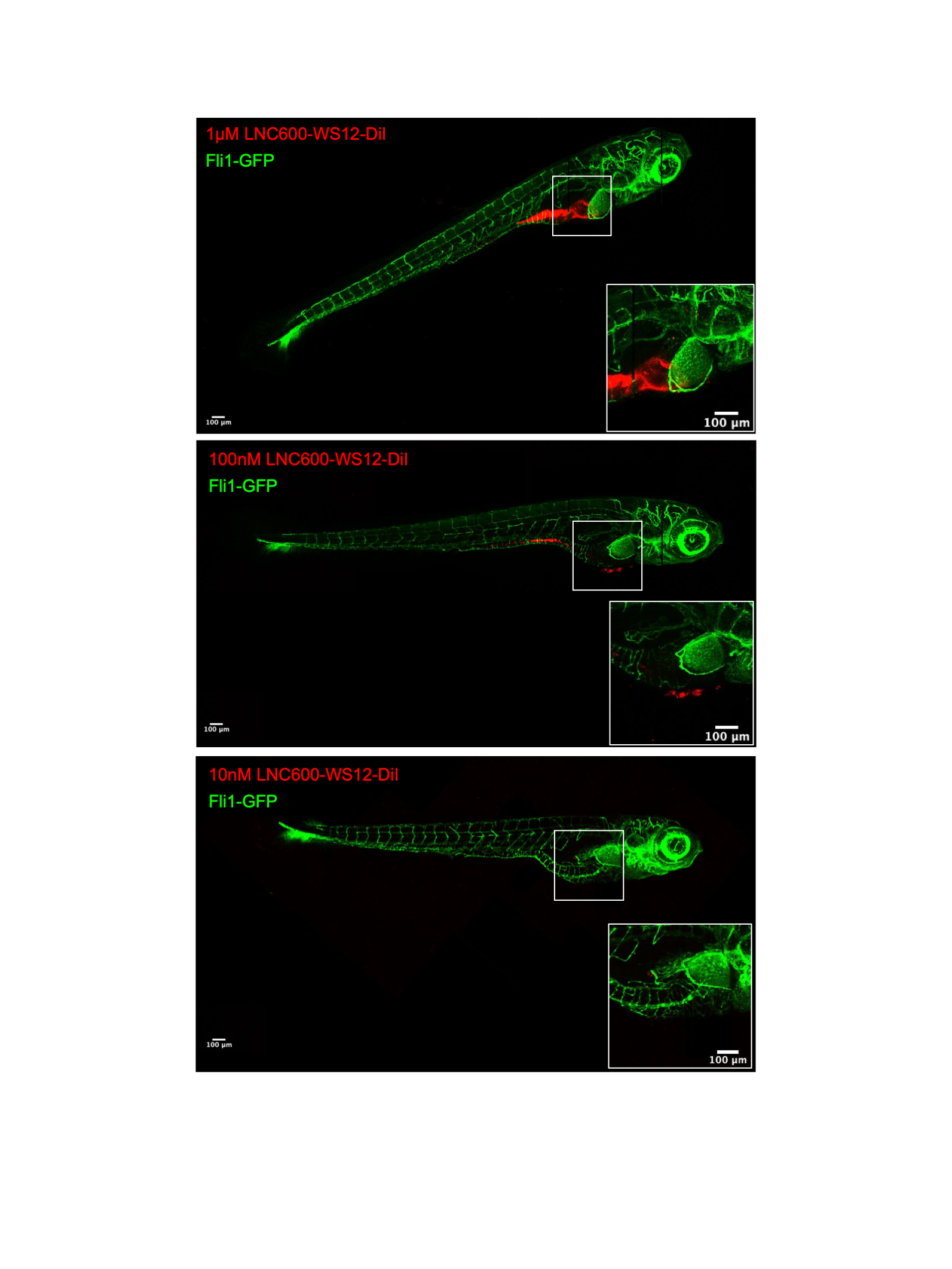
**

**Supplemental 3.** **Confocal analysis of LNC600-WS12 uptake in zebrafish.** LNC600-WS12 is labeled with DiI (red), and the results are shown for experiments performed in which 1 µM (**A**), 100 nM (**B**) or 10 nM (**C**) was used to treat zebrafish embryos for 6 days before the embryos were fixed. Confocal analysis showing the internalization of DiI-labeled LNC600-WS12 in the zebrafish. Zooms show the presence of LNC600-WS12 into the yolk sacs of zebrafish after treatment with 1µM and 100nM of LNC600-WS12.
